# Cerebrovascular Senescence Is Associated With Tau Pathology in Alzheimer's Disease

**DOI:** 10.3389/fneur.2020.575953

**Published:** 2020-09-16

**Authors:** Annie G. Bryant, Miwei Hu, Becky C. Carlyle, Steven E. Arnold, Matthew P. Frosch, Sudeshna Das, Bradley T. Hyman, Rachel E. Bennett

**Affiliations:** ^1^Department of Neurology, Harvard Medical School, MassGeneral Institute for Neurodegenerative Disease, Massachusetts General Hospital, Charlestown, MA, United States; ^2^Department of Pathology, Harvard Medical School, MassGeneral Institute for Neurodegenerative Disease, Massachusetts General Hospital, Charlestown, MA, United States

**Keywords:** Alzheimer's disease, tau pathology, neurofibrillary tangles, vascular dysfunction, endothelial senescence, gene expression, plasma biomarkers

## Abstract

Alzheimer's Disease (AD) is associated with neuropathological changes, including aggregation of tau neurofibrillary tangles (NFTs) and amyloid-beta plaques. Mounting evidence indicates that vascular dysfunction also plays a key role in the pathogenesis and progression of AD, in part through endothelial dysfunction. Based on findings in animal models that tau pathology induces vascular abnormalities and cellular senescence, we hypothesized that tau pathology in the human AD brain leads to vascular senescence. To explore this hypothesis, we isolated intact microvessels from the dorsolateral prefrontal cortex (PFC, BA9) from 16 subjects with advanced Braak stages (Braak V/VI, B3) and 12 control subjects (Braak 0/I/II, B1), and quantified expression of 42 genes associated with senescence, cell adhesion, and various endothelial cell functions. Genes associated with endothelial senescence and leukocyte adhesion, including SERPINE1 (PAI-1), CXCL8 (IL8), CXCL1, CXCL2, ICAM-2, and TIE1, were significantly upregulated in B3 microvessels after adjusting for sex and cerebrovascular pathology. In particular, the senescence-associated secretory phenotype genes SERPINE1 and CXCL8 were upregulated by more than 2-fold in B3 microvessels after adjusting for sex, cerebrovascular pathology, and age at death. Protein quantification data from longitudinal plasma samples for a subset of 13 (*n* = 9 B3, *n* = 4 B1) subjects showed no significant differences in plasma senescence or adhesion-associated protein levels, suggesting that these changes were not associated with systemic vascular alterations. Future investigations of senescence biomarkers in both the peripheral and cortical vasculature could further elucidate links between tau pathology and vascular changes in human AD.

## Introduction

Vascular dysfunction has become increasingly implicated in the pathogenesis of Alzheimer's Disease (AD) (1, 2). Cerebrovascular diseases including cerebral amyloid angiopathy (CAA) and atherosclerosis often co-present with AD (3–5), and reduced cerebral blood flow in the human AD brain is associated with both cognitive decline (6, 7) and tau accumulation (8–10). Proper function of the neurovascular unit, which is comprised primarily of endothelial cells, mural cells, and astrocytic endfeet, is essential for nutrient supply and protection from peripheral blood molecules via the blood-brain barrier (BBB). Impaired cerebral blood flow represents a key mechanism in neurodegeneration as it contributes to neuronal injury (11) and the accumulation of amyloid beta aggregates (12).

One of the neuropathological hallmarks of AD is the aggregation of phosphorylated tau protein into neurofibrillary tangles (NFTs). Several lines of research indicate that tau pathology may interact with endothelial cell changes to drive vascular impairment in AD. Senescence is a complex process in which cells irreversibly cease proliferating, change morphology, and present the senescence-associated secretory phenotype (SASP) (13). In neurons, cellular senescence and tau NFT formation have been reported to exhibit a positive feedback relationship (14, 15), and suppression of senescence prevents tau aggregation *in vivo* (15). Moreover, endothelial senescence is associated with vascular dysfunction via BBB disruption (16, 17), increased vascular stiffness (18, 19), and aberrant neurovascular coupling (10, 20–22).

The SASP profile includes pro-inflammatory cytokines, cell cycle regulators, and pro-angiogenic factors (13, 23, 24), which propagate senescence via autocrine and paracrine signaling (25, 26). This profile phenocopies, to some extent, changes we have observed in the microvasculature of tau-overexpressing transgenic (Tg4510) mice (27). We therefore examined whether endothelial senescence contributes to microvascular alterations in tau-related neurodegenerative disease. To investigate this hypothesis, we isolated intact microvessels from the dorsolateral prefrontal cortex (PFC, Brodmann area 9) of 16 AD and 12 control cases and measured expression of 42 genes related to senescence, cell adhesion, and general endothelial cell functions. Since many of the senescence-associated biomarkers are secreted, we also investigated whether changes in plasma protein levels could be detected in AD subjects in the years before death.

## Materials and Methods

### Subject Information

Twenty-eight cases were selected from the Neuropathology Core of the Massachusetts Alzheimer's Disease Research Center. Thirteen of the subjects also had longitudinal plasma sample collection over several years; these cases are indicated with an asterisk in the Plasma column in Table 1. All cases were assessed by a neuropathologist and scored for Alzheimer's Disease (AD) neuropathology burden according to the NIA-AA guidelines (28). Control cases (*n* = 12) were defined as subjects with a Braak neurofibrillary tangle (NFT) score of 0/I/II (B1) and were classified as low AD probability. One of the B1 subjects exhibited Lewy Body Disease with Primary Age-Related Tauopathy and one exhibited Progressive Supranuclear Palsy pathology, as labeled in Table 1. AD cases (*n* = 16) were defined as subjects with a Braak NFT score of V/VI (B3) indicating extensive tau pathology in the neocortex, with NIA-AA classification as intermediate or high AD probability. B3 subjects did not exhibit Lewy bodies, though 6 out of 16 had evidence of TDP-43 inclusions in the amygdala and hippocampus.

**Table 1:** Description of the 28 subjects included in this study.

**Subject ID**	**Plasma**	**ABC-Amyloid**	**ABC-Braak**	**ABC-CERAD**	**ABC-burden**	**Age at death**	**ApoE**	**Neuropathology notes**	**Cerebrovascular pathology**
AD01		2	3	2	Intermediate	80–90	e2/e3		
AD02		2	3	2	Intermediate	80–90	e3/e3		
AD03		3	3	1	Intermediate	>90	e4/e3		
AD04		2	3	2	Intermediate	80–90	e3/e3		CVD, INF
AD05		2	3	2	Intermediate	80–90	e3/e3		
AD06		2	3	2	Intermediate	80–90	e3/e3		
AD07	*	3	3	3	High	70–80	e4/e4		
AD08	*	3	3	3	High	60–70	e4/e3		
AD09	*	3	3	2	High	>90	e4/e3		CVD, CAA
AD10	*	3	3	2	High	80–90	e4/e3	Hippocampal Sclerosis	
AD11	*	3	3	3	High	80–90	e4/e4		
AD12		3	3	3	High	70–80	e4/e3		CVD
AD13	*	3	3	3	High	80–90	e4/e3		
AD14	*	3	3	2	High	80–90	e4/e3		CVD, CAA
AD15	*	3	3	3	High	60–70	e4/e3		CAA
AD16	*	3	3	2	High	>90	e2/e3		CVD, CAA
CTRL01		0	0	0	Not AD	50–60	e3/e3		
CTRL02	*	0	0	0	Not AD	>90	e3/e3	PSP	
CTRL03		0	1	0	Not AD	80–90	e3/e3		
CTRL04		0	1	1	Not AD	>90	e2/e3		
CTRL05		0	1	1	Not AD	>90	e3/e3		CVD
CTRL06	*	0	1	0	Not AD	>90	e3/e3		CVD, INF
CTRL07		0	1	0	Not AD	>90	e3/e3		
CTRL08		0	1	0	Not AD	70–80	e3/e3		
CTRL09	*	0	1	0	Not AD	>90	e3/e3		INF
CTRL10		2	1	1	Low	>90	e3/e3		CVD
CTRL11		1	1	1	Low	70–80	e3/e3		CAA
CTRL12	*	1	1	0	Low	>90	e3/e3	LBD, PART	CVD, Arteriolosclerosis

*These 28 subjects were selected for microvascular gene expression analysis. Age at death is binned by decade per journal policy. An asterisk in the “Plasma” column indicates the subject also had longitudinal plasma collection for protein quantification. PSP, progressive supranuclear palsy; LBD, Lewy-body dementia; PART, primary age-related tauopathy; CVD, cerebrovascular disease; INF, infarcts; CAA, cerebral amyloid angiopathy*.

### Cerebral Microvessel Isolation

We adapted this protocol for intact cortical microvessel isolation from Boulay et al. (29) and Hartz et al. (30), which is designed to protect the intercellular connections between endothelial cells and mural cells. All steps for microvessel isolation were performed on ice under RNAse-free conditions. Approximately 200 mg of frozen postmortem dorsolateral prefrontal cortex (PFC, Brodmann area 9) tissue was measured by a histologist for each case. Meninges were removed from the cortical surface and the cortical tissue was sliced into ~2 mm sections using a sterile razor blade in Hank's Balanced Salt Solution (HBSS) and HEPES buffer. The tissue sections were manually dissociated with a dounce homogenizer and centrifuged for 10 min at 2,000 g in 4°C. The pellet was resuspended in a dextran solution and centrifuged at 4,400 g for 15 min at 4°C. A myelin layer formed atop the supernatant, which was removed by inverting the centrifuge tube and carefully blotting the inside walls. The pellet was resuspended in the HBSS-HEPES buffer with RNAse-free bovine serum albumin (BSA) and filtered over a 20 μm mesh filter (Millipore) to collect microvessels. Microvessels were collected from the filter by gently stirring the filter in HBSS-HEPES-BSA buffer. The collected microvessels were washed and spun twice at 2,000 g for 5 min at 4°C. The supernatant was discarded and the pellet containing isolated microvessels was stored at −80°C prior to RNA isolation.

### RNA Isolation and RT-qPCR

Frozen microvessel pellets or 25 mg of whole tissue was resuspended in RLT buffer (Qiagen) and sonicated for 20 pulses at 10% amplitude to lyse cells. The lysed cells were then centrifuged at 13,000 rpm for 3 min to pellet out residual cellular debris. RNA was extracted using the RNeasy Mini Kit (Qiagen) and was eluted in 30 μl of nuclease-free water. RNA was assessed using the NanoDrop spectrophotometer (ThermoFisher) and diluted to a standard concentration of 20 ng/μl for each sample. cDNA was synthesized using the RT^2^ HT First Strand kit (Qiagen) and was combined with RT^2^ SYBR Green Mastermix fluorescent dye (Qiagen). The cDNA reaction mix was loaded into a custom 96-well RT^2^ Profiler Array with pre-loaded primers. Each plate contained 42 genes of interest (Table 2), three housekeeping genes, a Human Genomic DNA Control (HGDC), a Reverse Transcription Control (RTC), and a Positive PCR Control (PPC). Two samples were run per plate. The plate was covered with a plastic seal to prevent sample evaporation and briefly spun at ~400 rpm to remove bubbles. The qPCR reactions were performed using the BioRad CFX96 Real-Time Detection System. The sequence began with a 10-min incubation at 95°C to activate the DNA polymerase enzyme. Fluorescence data collection then commenced with 40 cycles of alternating 15 s at 95°C and 60 s at 60°C.

**Table 2:** Gene and protein biomarkers analyzed in this study.

**Hypothesis-driven functional group**	**Description**	**References**	**Genes** **(*n* = 42)**	**Proteins** **(*n* = 22)**
Senescence-associated	The endothelial senescence-associated secretory phenotype (SASP), a suite of inflammation- and angiogenesis-associated genes upregulated in endothelial senescence.	(13, 23, 24, 31)	CDKN1A	
			CDKN2A	
			CSF2	
			CXCL1	CXCL1
			CXCL2	
			CXCL8	IL8
			IL6	IL6
			SERPINE1	PAI-1
Cell adhesion molecules	These genes encode cell adhesion proteins active in the cerebrovascular endothelium to mediate endothelial-leukocyte adhesion, a process central to the inflammatory response.	(32–34)	EMCN	
			ICAM1	ICAM-1
			ICAM2	ICAM-2
			MAdCAM1	
			SELE	SELE
			SELP	SELP
			VCAM1	VCAM1
Endothelial cell markers	These genes are specifically expressed in endothelial cells and facilitate various endothelial cell functions.	(35–42)	NOSTRIN	
			PECAM1	PECAM1
			SLC2A1	
			TEK	TIE2
			TIE1	TIE1
			VWF	VWF
Junction proteins	These genes encode proteins that are integral to the formation of endothelial gap junctions and tight junctions, which mediate vasodilation and enable strict regulation of molecular transport across the BBB.	(43–45)	CDH5	CDH5
			CLDN5	
			ESAM	
			GJA1	
			OCLN	
			TJP1	
VEGF/Notch pathway	These genes encode part of an angiogenesis-regulating protein network that exhibits aberrant expression in the AD cerebral vasculature.	(46–50)	ADGRL4	
			DLL4	
			ENG	ENG
			FLT1	
			KDR	VEGFR-2
			VEGFA	VEGFA
Cell stress	HMOX1 and NOS3 are upregulated in endothelial cells in response to cell stressors including oxidative stress and hypoxia.	(51, 52)	HMOX1	HO-1
			NOS3	NOS3
Plasmin/APOE pathways	In this pathway, LRP1 binds the plasmin activators tPA (encoded by PLAT) and uPA (encoded by PLAU), as well as APOE, for internalization and proteolysis. The regulation of this system is implicated in BBB integrity and proper clearance of amyloid-beta.	(53–55)	APOE	
			LRP1	
			PLAT	tPA
			PLAU	uPA
Other cell markers	ALDH1L1, ACTA2, and ITGAM (aka CD11b) are putative markers of astrocytes, smooth muscle cells, and microglia, respectively.	(56–58)	ALDH1L1	
			ITGAM	ITGAM
			ACTA2	

*The roles of these genes and proteins were classified into hypothesized functional groups according to potential contributions to vascular dysfunction in the human AD brain, with corresponding references. Gene and corresponding protein names are listed*.

The ΔΔCT relative quantification method was used to calculate gene expression (59). First, positive controls for reverse transcription and PCR (RTC and PPC) were analyzed for each sample according to manufacturer instructions. Gene Ct (cycle threshold) results were quality filtered to remove any data points with a Ct value greater than the sample Ct for the genomic DNA (HGDC) control primer. Any reaction with no fluorescence detection by 40 qPCR cycles was reported as not detected, and any gene with undetectable expression in more than 25% of samples in total was excluded. Three reference genes were included in the RT^2^-Profiler array: ACTB, GAPDH, and PGK1. The geometric mean of the reference gene Ct values was calculated for each subject and subtracted from the Ct value of each target gene, yielding ΔCT values. For each gene, the average ΔCT value in B1 subjects was then subtracted from each sample's ΔCT value to obtain ΔΔCT values. The relative quantification (RQ) value was calculated as 2^−ΔΔCT^, which was then log2-transformed to yield fold changes.

Cell-type specific genes were included to verify the presence of vascular cells in microvessel isolates and compared to bulk RNA measures from total cortex. Using the endothelial cell marker PECAM1 (CD31) and the vascular smooth muscle cell marker ACTA2, we confirmed these cells are significantly enriched in isolated microvessels compared to total cortex homogenate in 16 representative samples (Supplementary Figure 1A). Astrocytes (ALDH1L1) were not enriched in isolated microvessels compared with total cortex, and microglial (ITGAM) cell composition was not statistically different. There was also no statistical difference in cell composition between B1 vs. B3 microvessels when examining all *n* = 28 microvessel samples (Supplementary Figure 1B). Overall, endothelial cell and vascular smooth muscle cell expression was greater than astrocyte and microglia expression in isolated microvessels.

### Plasma Proteomics

For 13 of the subjects (*n* = 4 B1, *n* = 9 B3), plasma was collected at two or three time points spaced at ~1-year intervals (Table 1, labeled with asterisks). Plasma was sent to Olink Proteomics for protein quantification using 92-plex antibody labeling with a proximity extension assay. Quantification results were normalized and log-2 transformed and were reported as Normalized Protein eXpression (NPX) values. Twenty-two of the biomarkers analyzed via RT-qPCR were also included in the Olink protein panels (Table 2). Quality control was applied by eliminating any data points with an NPX value below the manufacturer-specified protein limit of detection (LOD) threshold.

### Statistical Analysis

All statistical analyses and data visualizations were performed with R (v4.0.0). A significance level was set at *p* < 0.05, and correction for multiple comparisons was applied where necessary using the Benjamini-Hochberg (BH) False Discovery Rate (FDR) (60). This was calculated with the p.adjust() function in R using the “stats” package (v4.0.0).

Univariate comparisons were conducted using Welch's two-way unpaired *t*-test using the t.test() function from the R “stats” package. To test the robustness of a given result, bootstrapped iterations were performed in which 75% (12/16) of B3 and 83% (10/12) of B1 subjects were randomly sampled 1,000 times, and the sample estimates were computed (61). The average fold change difference and proportion of significant iterations (*p* < 0.05) were reported to summarize bootstrapped iteration results. Regression models were implemented using the lm() function from the R “stats” package and results were reported using the tidy.lm() function from the “broom” R package (v0.5.6). ANOVA and Tukey's *post-hoc* HSD test were implemented using the aov() functions from the R “stats” package and the tukey_hsd() function from “rstatix” (v0.6.0). Note: Missing data were excluded on a gene-wise basis for *t*-test and regression analyses, though imputation with the highest Ct value per gene was also examined (62).

Principal component analysis (PCA) was implemented using the “FactoMineR” (v2.3) and “factoextra” (v1.0.7) packages in R. As PCA requires complete data, missing data were imputed on a gene-wise basis with the highest observed Ct value for the corresponding gene (62). Fold change data were centered to have a mean of zero before PCA was applied using the prcomp() function in FactoMineR. The proportion of variance per principal component (PC) as well as cumulative variance were examined with a Scree plot to determine how many components would be further analyzed.

## Results

### Senescence-Associated Genes Are Upregulated in B3 PFC Microvessels

We isolated intact microvessels from the dorsolateral prefrontal cortex (PFC, BA9) to examine gene expression changes related to the extent of AD-related tau pathology. Enrichment of vascular transcripts in microvessel preparations was first verified by RT-qPCR (see Methods, Supplementary Figure 1). The PFC was selected as a Braak III/IV (B2) region, meaning it does not generally exhibit tau NFT pathology in early-stage AD (63). Braak 0/I/II (B1) subjects are therefore unlikely to present any PFC tau pathology, while Braak V/VI (B3) subjects are likely to have a high tau NFT burden in the PFC (64).

**Figure 1:**
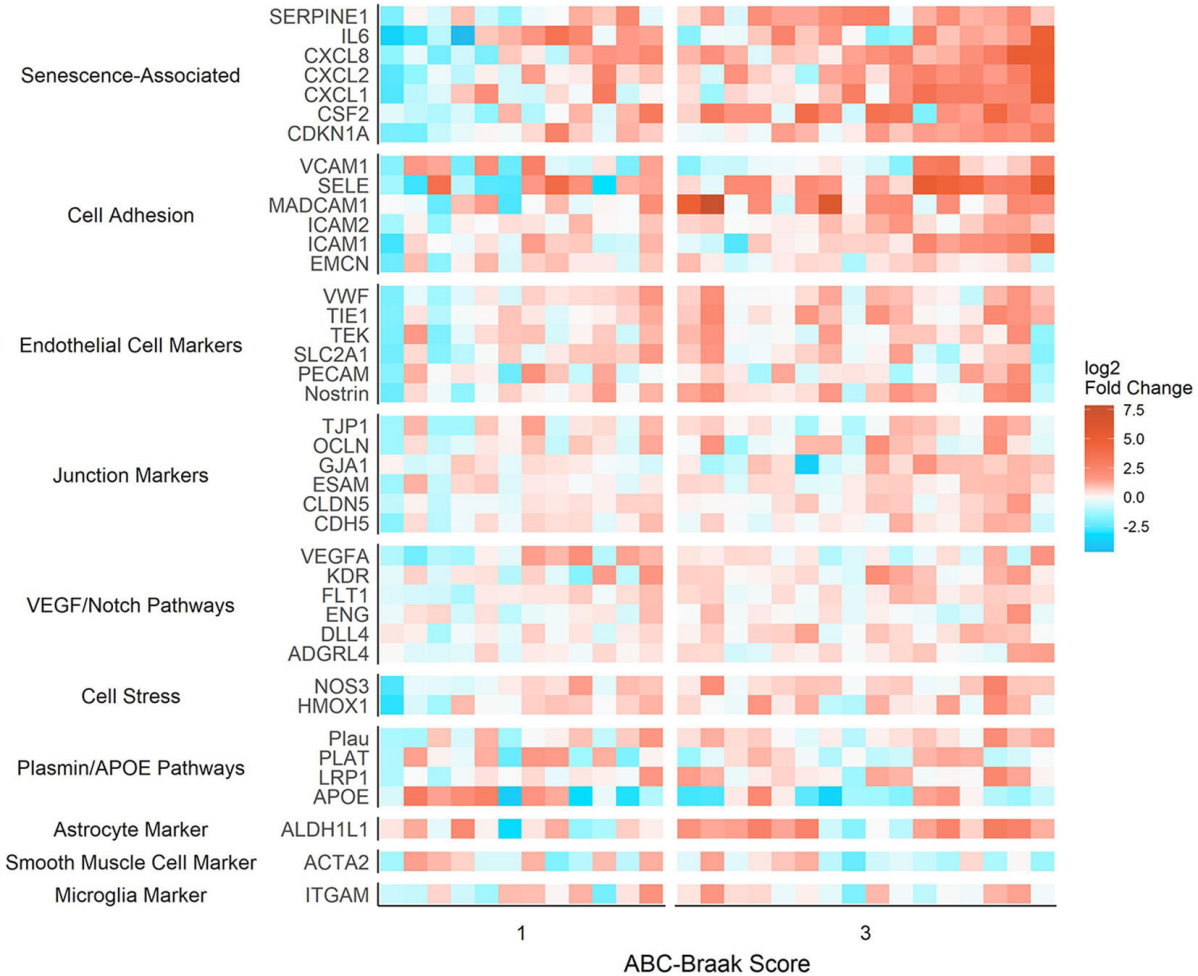
Senescence-associated genes form a fold change hotspot in prefrontal cortex microvessels isolated from high-Braak (B3) vs. low-Braak (B1) samples. Differential gene expression between microvessels isolated from B3 vs. B1 samples, grouped by hypothesis-driven gene function groups. Values reflect log2-transformed fold change values relative to the B1 subject mean expression per gene. Relative quantification was performed such that the mean of B1 fold changes is zero for each gene. For this visualization, missing data were imputed with the ABC-Braak group mean fold change for the gene.

**Figure 2:**
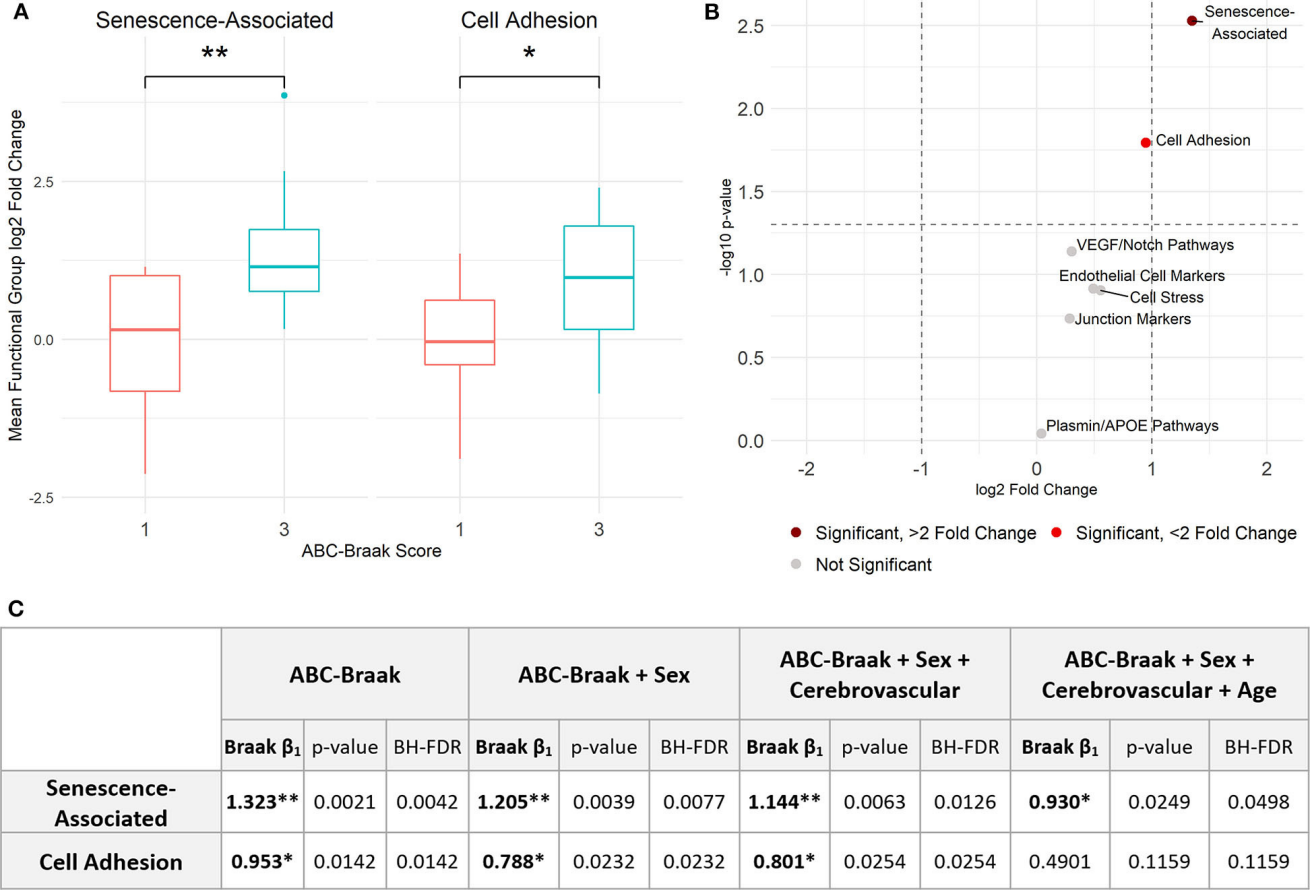
Senescence- and adhesion-associated genes are significantly upregulated in prefrontal cortex microvessels isolated from B3 vs. B1 subjects. **(A)** Senescence-associated genes are significantly upregulated in B3 cerebral microvessels with a log2 fold change of 1.323; Welch's unpaired *t*-test, *p* = 0.0030 (BH-corrected FDR 0.0208), 95% CI for B3 log2 fold change: 0.5019, 2.1442. Cell adhesion genes are also significantly upregulated with a log2 fold change of 0.953; Welch's unpaired *t*-test, *p* = 0.0161 (BH-FDR 0.0562), 95% CI for B3 log2 fold change: 0.1945, 1.7123. **(B)** Volcano plot showing *t*-test results. Dashed vertical lines denote magnitudes of 2-fold change and the dashed horizontal line denotes the cutoff for *p* < 0.05. **(C)** Stepwise multiple regression models were applied to senescence-associated and cell adhesion functional groups to measure the association between Braak stage and functional group average fold change. The first row indicates the independent variables upon which the average fold change was regressed per model for each functional group. Within each model, the ABC-Braak term regression coefficient (β_1_), *p*-value, and BH-FDR are reported. Significant ABC-Braak β1 terms are denoted with **p < 0.01, *p < 0.05.

To assess gene expression associated with senescence, cell adhesion, and endothelial cell function, we measured expression of 42 genes classified into eight hypothesis-driven functional groups (Table 2). These eight classes were proposed based on literature reviewed in Table 2. Of the 42 genes measured, CDKN2A and SELP were excluded due to undetectable expression in more than 25% of samples (defined as no fluorescence detection by 40 cycles or a Ct value above the Human Genomic DNA Contamination Ct value) (Supplementary Figure 2). For the remaining 40 genes, heatmap visualization of log2 fold changes showed an observable hotspot of senescence-associated gene upregulation in B3 PFC microvessels (Figure 1). The average functional group fold change value was calculated per subject (excluding the non-endothelial cell markers) which confirmed a 2.5-fold increase in senescence-associated gene mRNA in B3 microvessels (Figure 2A; *p* = 0.0030, BH-FDR = 0.0208, 95% CI: [0.5019, 2.1442]). Additionally, expression of cell adhesion genes was increased by 1.9-fold in B3 microvessels compared to B1 microvessels (Figure 2A; *p* = 0.0161, BH-FDR=0.0562, 95% CI: [0.1945, 1.7123]). Missing values were omitted from these mean fold change calculations, though very similar results were obtained by imputing missing values with the highest Ct value of the gene. The other functional groups examined did not exhibit differential expression in B1 vs. B3 PFC microvessels (Figure 2B).

Sex, cerebrovascular pathology, and age can contribute to AD neurodegeneration, both directly and via tau pathology (65). To adjust for these important and potentially confounding covariates, stepwise multiple regression models were implemented to compare senescence-associated and cell adhesion mean fold changes between B3 vs. B1 samples (Figure 2C). We created the binary variable “Cerebrovascular pathology” to indicate presence or absence of at least one of cerebral amyloid angiopathy (CAA), cerebrovascular disease (CVD), and infarcts in the subject's neuropathology report (Table 1). Senescence-associated gene expression was still significantly increased in B3 microvessels compared to B1 after adjusting for sex, cerebrovascular pathology, and age. Cell adhesion gene expression was significantly increased in B3 microvessels after adjusting for sex and cerebrovascular pathology but not age at death, indicating that microvascular adhesion gene expression may be related to age. We note there was no statistical difference in the age at death between B1 and B3 subjects (Supplementary Figure 3).

### IL-8, PAI-1, and TIE1 Demonstrate Robust Upregulation in B3 PFC Microvessels Independent of Age, Sex, and Cerebrovascular Pathology

**Figure 3:**
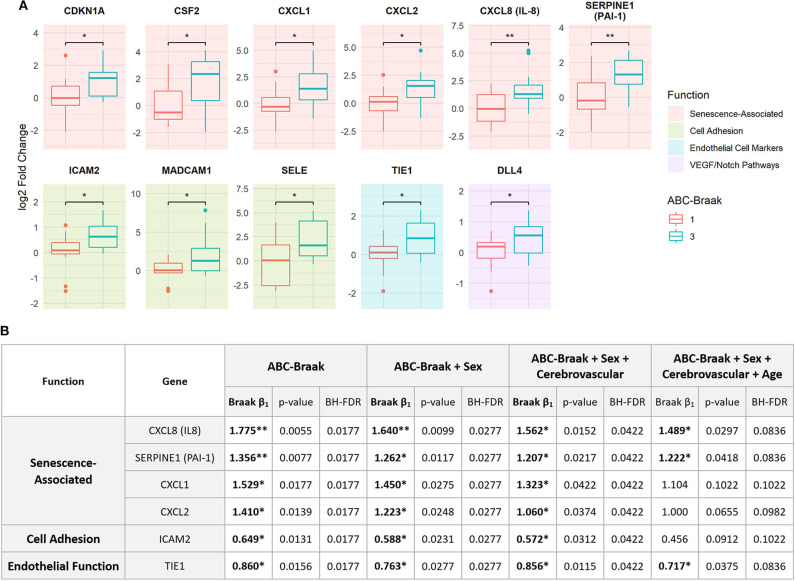
Eleven genes related to senescence, adhesion, endothelial cell function, and VEGF/NOTCH pathways are significantly upregulated in PFC capillaries from B3 samples relative to B1 samples. **(A)** 11 genes show significant upregulation in B3 vs. B1 microvessels. Welch's unpaired *t*-test results are shown: **p < 0.01, *p < 0.05, all BH-FDR < 0.2. Plots are shaded by hypothesis-driven functional group. **(B)** Stepwise multiple regression models were applied to each of the 6 most-consistently upregulated genes to measure the association between Braak change and log2 fold change, adjusting for potential confounders. The first row indicates the independent variables upon which the log2 fold change was regressed per model for each gene. Within each model, the ABC-Braak term regression coefficient (β_1_), *p*-value, and BH-FDR are reported. Significant ABC-Braak β1 terms are marked in bold with **p < 0.01, *p < 0.05.

After observing the general upregulation of senescence-associated and cell adhesion functional group genes in B3 PFC microvessels, we next investigated changes in individual genes. Of the 40 genes that we measured in PFC microvessels which passed quality control filtering, 11 were significantly upregulated (with *p* < 0.05, BH-FDR <0.20): CXCL8 (IL8), SERPINE1 (PAI-1), CXCL1, CXCL2, CSF2, CDKN1A, ICAM-2, MAdCAM-1, SELE, TIE1, and DLL4 (Figure 3A). With the exception of CDKN1A, all of the senescence-associated genes in this group exhibited greater than a 2-fold average increase in mRNA expression in B3 microvessels. Further, these results were robust for CXCL8 (IL-8), SERPINE1 (PAI-1), CXCL1, CXCL2, TIE1, and ICAM-2, which were significantly upregulated (*p* < 0.05) in 62–94% of 1,000 bootstrapped iterations (Supplementary Figure 4). Missing values were excluded on a gene-wise basis for these comparisons, though these six genes were still significantly upregulated after imputing missing values with the highest Ct value of the gene.

For each of these six robustly upregulated genes, individual linear models were fit to regress gene fold change on ABC-Braak score, with stepwise inclusion of sex, cerebrovascular pathology, and age as covariates. The increased expression in B3 samples remained significant for all six genes after adjusting for sex and cerebrovascular pathology, indicating their upregulation was independent of these factors (Figure 3B). However, with the inclusion of age at death in the regression model, only CXCL8 (IL-8), SERPINE1 (PAI-1), and TIE1 were still significantly upregulated in B3 microvessels, suggesting their upregulation was age-independent. For the other three genes (ICAM-2, CXCL1, CXCL2), elevated expression in B3 PFC microvessels may be associated with age.

### Tau Pathology and Cerebrovascular Pathology Interact to Increase Microvessel MAdCAM-1 Expression

**Figure 4:**
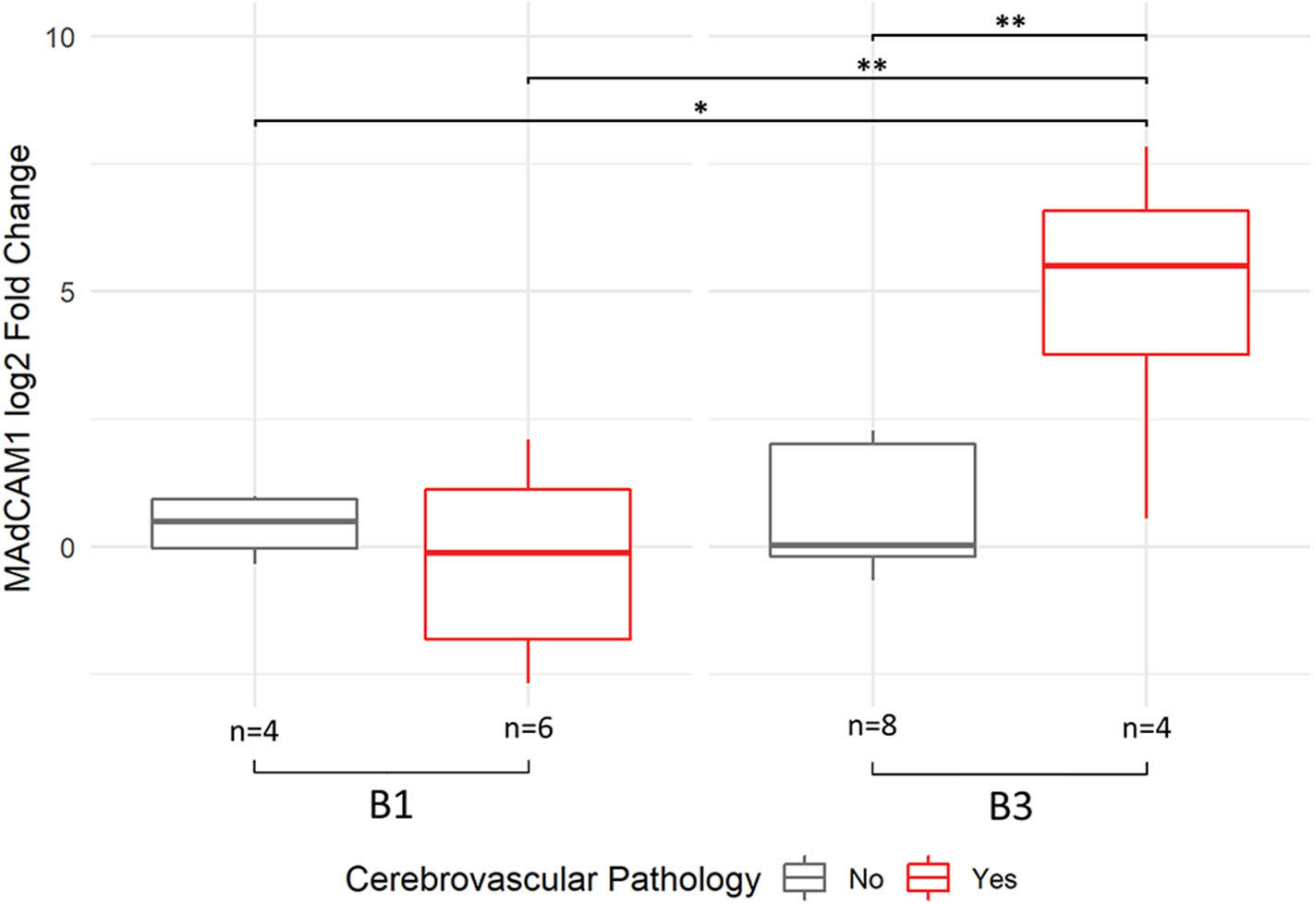
MAdCAM-1 upregulation demonstrates interaction between Braak stage and presence of cerebrovascular pathology. ANOVA with interaction demonstrated significant interaction between a high Braak score (B3) and presence of cerebrovascular pathology in MAdCAM1 expression out of all 40 genes quantified. Data shown in the boxplot reflect Tukey's HSD *post-hoc* test results, with **p < 0.01, *p < 0.05. A total of 6 samples did not have detectable expression, with an even distribution of missing data across the four groups. The number of samples with detected MAdCAM-1 expression per group are indicated at the bottom of the boxes.

Approximately half of both the B1 and B3 subjects exhibited at least one form of cerebrovascular pathology (Table 1), which is consistent with population-based studies in elderly demented and non-demented individuals (66, 67). Given the documented association between tau pathology and cerebrovascular pathology (68–70), we investigated whether the two interact in association with microvessel gene expression. Two-way ANOVA revealed an interaction between tau pathology and cerebrovascular pathology in MAdCAM-1 expression in PFC microvessels, which was significant prior to adjusting for multiple comparisons (Supplementary Figure 5A). *Post-hoc* analysis via Tukey's HSD test revealed that MAdCAM-1 mRNA expression was upregulated by more than 16-fold (4 log2-fold) in B3 subjects with cerebrovascular pathology compared to all other subjects (Figure 4, Supplementary Figure 5B). Of note, the top three MAdCAM-1 expression values correspond to B3 subjects with concomitant cerebral amyloid angiopathy (CAA) and general cerebrovascular disease (CVD). However, we note that the two-way ANOVA was not significant for MAdCAM-1 when missing values were imputed with the highest MAdCAM-1 Ct value.

### Endothelial Senescence-Associated and Cell Adhesion Gene-Driven Principal Component Composite Score Distinguishes B3 vs. B1 Microvessels

**Figure 5:**
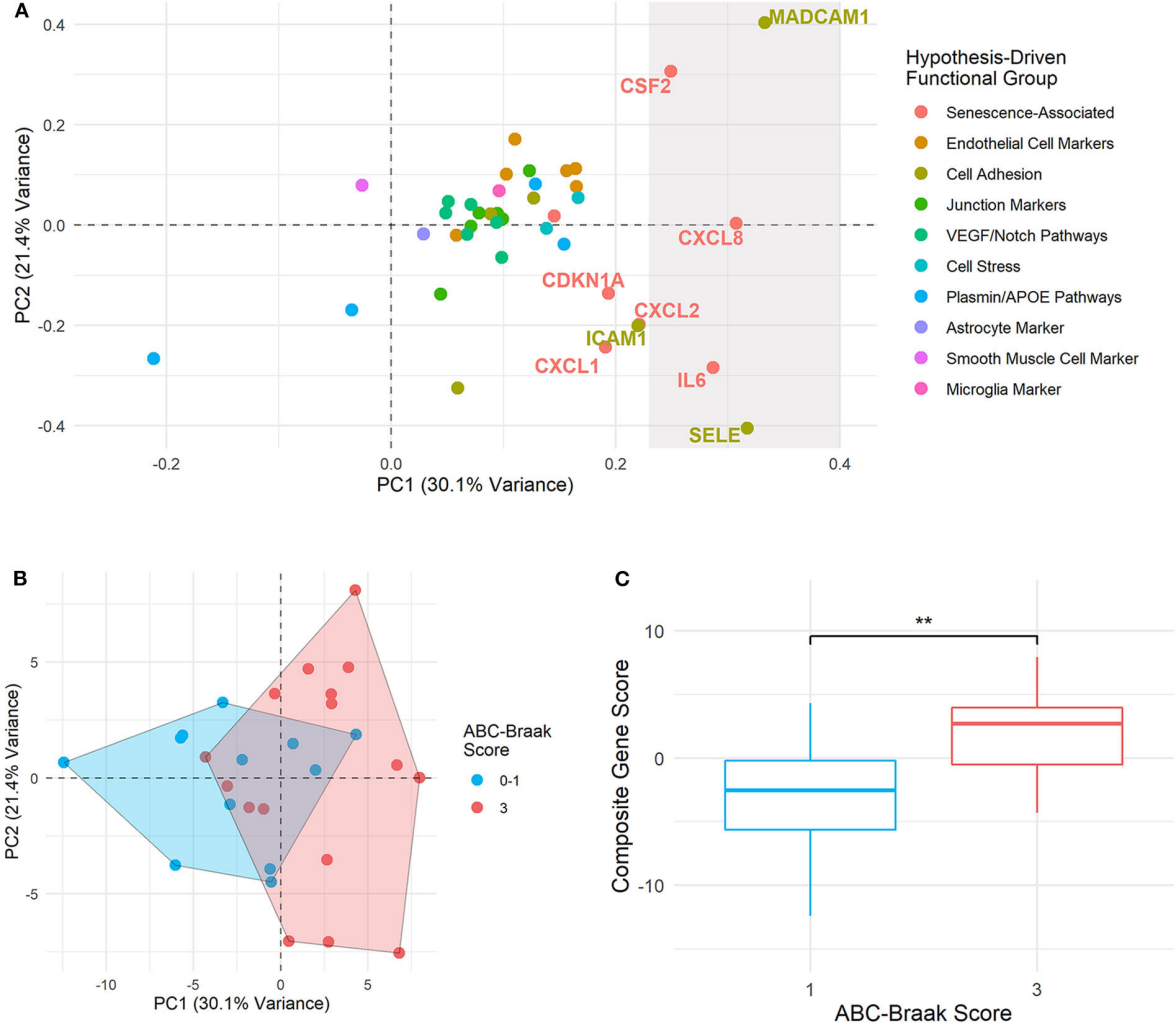
Principal component composite gene scores in B3 vs. B1 microvessels show variance driven by senescence and adhesion genes. **(A)** PC loadings are plotted for each gene in PC1 (x-axis) and PC2 (y-axis). The rightmost shaded region contains the genes considered to be top contributors to PC1, with their percent contribution at least one standard deviation above the mean contribution. Genes immediately adjacent to this top contributor subset are also labeled. Point and text colors correspond to each gene's hypothesis-driven functional group, listed on the right. **(B)** Samples are plotted by PC1 and PC2 composite gene fold change scores, with geometric encircling to outline B1 vs. B3 group boundaries. **(C)** Boxplots show PC1 composite gene fold change scores in B1 vs. B3 PFC microvessels. Welch's unpaired *t*-test was applied to compare B1 and B3 composite scores, with ** indicating *p* < 0.01. Composite gene fold change scores are significantly larger in B3 vs. B1 microvessels (*p* = 0.0064, 95% CI: [1.477, 7.956]).

To explore patterns of gene covariance in B1 vs. B3 cortical microvessels, principal component (PC) analysis was applied to centered fold change values for all 40 genes. The first two PCs collectively explained 51.2% of variance across the samples (Supplementary Figure 6). The top contributors to the first principal component (PC1), defined as those with more than one standard deviation above the mean contribution, included senescence-associated genes (IL6, CXCL8, CSF2) and cell adhesion genes (SELE and MAdCAM-1) (Figure 5A). As these genes are associated with endothelial dysfunction and/or senescence (23, 24, 31, 71–73), we hypothesized that PC-derived composite gene expression scores (a.k.a. PC composite score) would distinguish B3 from B1 microvessels. Indeed, the PC scores from the first and second principal components show partial separation of the B1 and B3 sample clusters, primarily along the PC1 axis (Figure 5B). This difference was statistically significant for the PC1 composite score, with B3 samples exhibiting significantly larger composite scores (Welch's unpaired *t*-test, *p* = 0.0064, 95% CI: [1.477, 7.956]; Figure 5C).

Four of the five genes driving fold change composite scores—CXCL8 (IL8), CSF2, SELE, and MAdCAM-1—were identified as upregulated in B3 microvessels, with CXCL8 (IL8) upregulation particularly pronounced after adjusting for age, sex, and cerebrovascular pathology (Figure 3). By contrast, IL6 upregulation did not reach significance in B3 microvessels. Its strong contribution to the gene composite score that separated B1 vs. B3 microvessels may indicate that IL-6 is associated with ABC-Braak differences in the context of other senescence-associated and cell adhesion genes. Of note, IL6 expression differences were not driven by any other neuropathological variable, sex, APOE genotype, or cerebrovascular pathology (Supplementary Figure 7). Furthermore, PCA composite scores did not improve separation B1 vs. B3 microvessels based on these other covariates relative to ABC-Braak scores (Supplementary Figure 8).

### NOS3 Antemortem Plasma Protein Expression Is Associated With Postmortem Cortical Microvessel Gene Expression

The cerebral vasculature interacts with the peripheral vasculature via plasma proteins and soluble blood factors (74). Therefore, we compared postmortem PFC microvessel gene expression with antemortem plasma protein expression in a subset of 13 subjects with both metrics available (Table 1, subjects with asterisks). In total, 22 proteins overlapped with cognate genes measured in our qPCR assay (Table 2), though SELP was not analyzed as it was excluded from qPCR analysis due to a high proportion of missing data. Protein expression was quantified as Normalized Protein eXpression (NPX), a log2-transformed unit that enables inter-subject comparison within a given protein. Of note, two ITGAM values were omitted as they were below the manufacturer-specified limit of detection (see Methods). Protein expression was measured longitudinally in two or three plasma samples per subject, though we focused here on the final plasma samples to minimize pre-mortem intervals.

**Figure 6:**
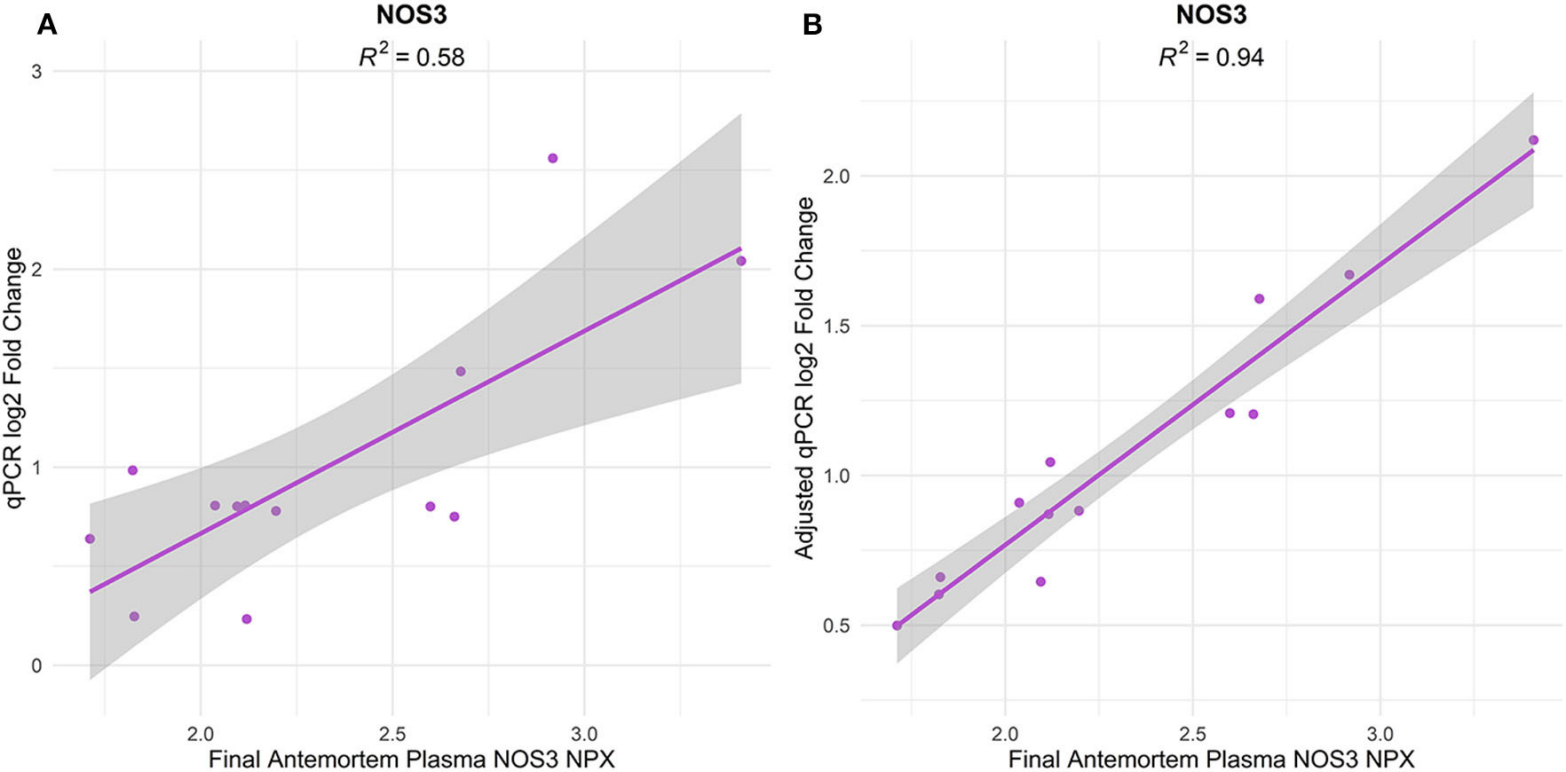
NOS3 antemortem plasma expression is significantly associated with postmortem NOS3 gene expression in PFC capillaries of both B1 and B3 subjects. **(A)** Univariate regression of NOS3 plasma NPX (from final plasma sample per subject) vs. postmortem microvessel gene log2 fold change demonstrated a significant association between NPX and log2 fold change (β1 =1.022, *p* = 0.0024, BH-FDR = 0.0511). The shaded region indicates the 95% confidence interval of the regression slope. **(B)** After adjusting for years until death in multiple regression, there is still a significant association between NOS3 plasma NPX and postmortem capillary gene log2 fold change (β1 = 1.070, *p* = 0.0031, BH-FDR = 0.0660). For visualization, the adjusted log2 fold change values were calculated as the NPX β1 coefficient plus the partial residual associated between plasma NPX and years until death.

Univariate regression revealed that NOS3 postmortem microvessel expression was significantly associated with antemortem plasma expression (Figure 6A; NPX β_1_ = 1.022, *p* = 0.0024, BH-FDR = 0.0511, *R*^2^ = 0.58). However, the pre-mortem interval ranged from 1 month to 6.7 years among samples. To account for this variability, we included pre-mortem interval as a covariate in a subsequent multiple regression. This still yielded a significant linear relationship between NOS3 plasma NPX and PFC microvessel gene fold change (NPX β_1_ = 1.070, *p* = 0.0031, BH-FDR = 0.0660). For visualization, the gene fold change values were adjusted for the partial residual of the model, which demonstrated a strong linear association between antemortem plasma NPX and postmortem gene expression (*R*^2^ = 0.94, Figure 6B).

### Plasma Senescence-Associated Protein Levels Are Not Associated With Postmortem Severity of AD-Related Neuropathological Changes

**Figure 7:**
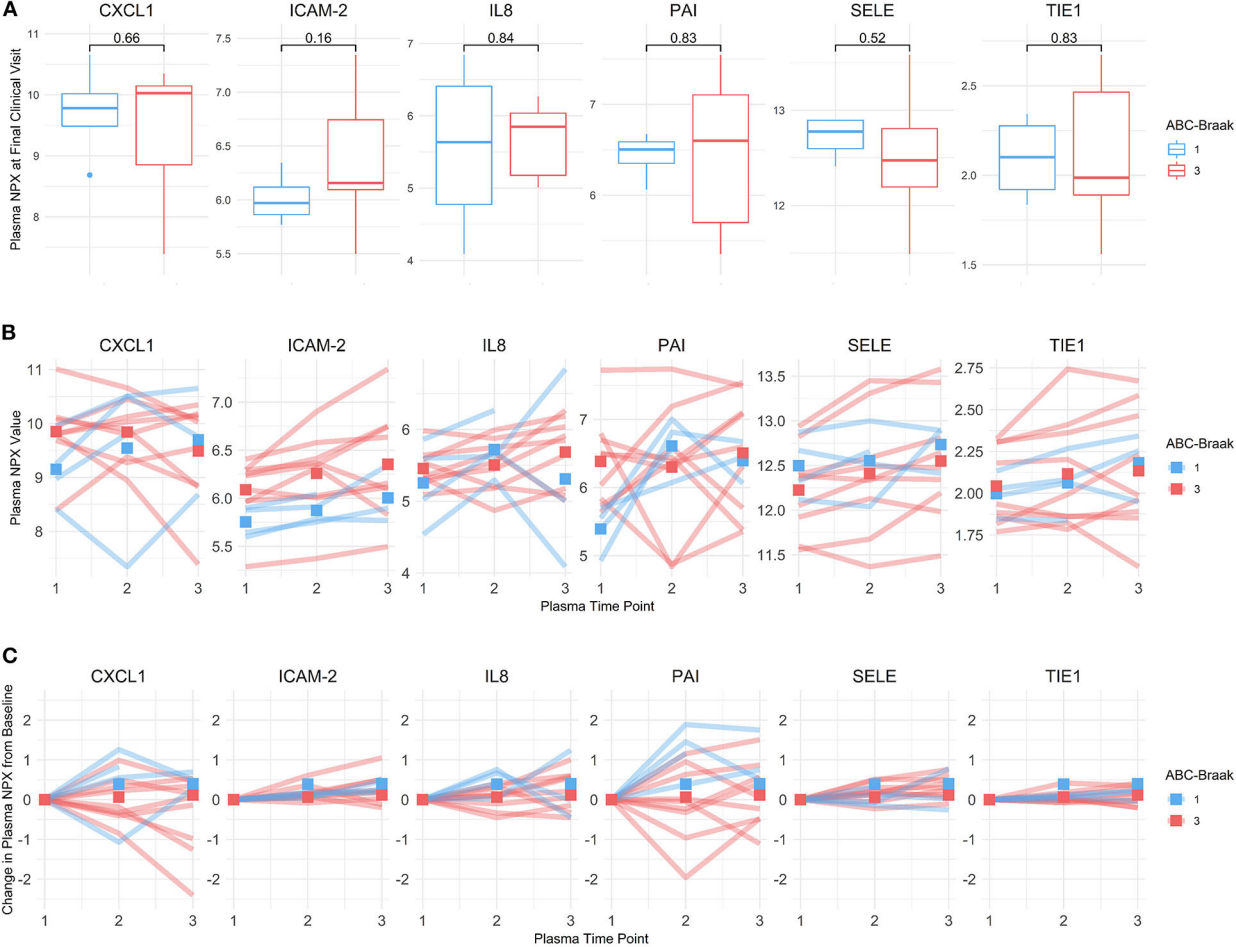
B1 vs. B3 subject plasma expression of protein biomarkers corresponding to genes upregulated in B3 cortical PFC microvessels. **(A)** NPX values from each subject's last plasma sample for CXCL1, ICAM-2, IL8, PAI-1, SELE, and TIE1. B1 samples are shown in blue and B3 samples are shown in red. Welch's unpaired *t*-test *p*-values are shown above the boxes. **(B)** Plasma NPX values across three longitudinal samples per subject for CXCL1, ICAM-2, IL8, PAI-1, SELE, and TIE1. B1 samples are shown in blue and B3 samples are shown in red. Squares represent the ABC-Braak group mean NPX value at the corresponding plasma sample time point (which is an arbitrary unit, relative to each subject). **(C)** Relative plasma NPX change from baseline for CXCL1, ICAM-2, IL8, PAI-1, SELE, and TIE1. Values on the y-axis represent net change in plasma NPX from the first plasma sample, which is set to zero for all subjects for visualization. Squares represent the ABC-Braak group mean change in NPX at the corresponding plasma sample time point. Scales are fixed for all six proteins to compare relative temporal stability.

Six of the genes that were upregulated in B3 microvessels were also quantified in plasma as secreted proteins: IL8 (CXCL8), PAI-1 (SERPINE1), CXCL1, TIE1, ICAM-2, and SELE. We reasoned that the changes seen in the CNS microvessels might reflect systemic vascular factors, which might then be observed in the plasma; alternatively, CNS-only microvascular changes may be difficult to detect in the systemic circulation. We therefore investigated whether these biomarkers exhibited protein expression differences in B1 vs. B3 antemortem plasma. Univariate analysis comparing the protein NPX from the final plasma sample between B1 vs. B3 samples failed to yield any significant results (Figure 7A) as measured via *t*-test and linear regression. Similarly, multivariate analysis adjusting for sex, age at visit, and pre-mortem interval yielded no significant differences in protein NPX by Braak score. While the magnitude of NPX measurements cannot be directly compared between proteins, their relative changes from baseline can be compared to assess temporal stability. Plasma time points differed between subjects, with an average of 1.30 years (±0.38) between plasma samples per subject. To that end, PAI-1 levels exhibited the greatest fluctuations from baseline across all subjects, followed by CXCL1 (Figures 7B,C). By contrast, TIE1 plasma levels remained the most stable over time across all subjects.

None of the other 15 plasma proteins assayed in this study showed statistically different plasma levels in B1 vs. B3 subjects. Further descriptive and exploratory analyses were carried out to examine effects of age and pre-mortem interval duration on plasma protein levels. In a multiple regression of plasma protein NPX on ABC-Braak, age at visit, and pre-mortem interval duration, IL6, IL8, and ITGAM exhibited negative slopes between pre-mortem interval vs. plasma NPX before adjusting for multiple comparisons (Table 3). This indicates that subjects with longer pre-mortem intervals generally had lower IL6, IL8, and ITGAM plasma protein levels. IL6 also exhibited a positive slope between age vs. plasma NPX, as did CXCL1, suggesting that IL6 and CXCL1 plasma secretion may increase with age.

**Table 3:** Age and pre-mortem interval significantly influence plasma NPX expression.

**Protein**	**Regression term**	**β**_**1**_	***p*-value**	**BH-FDR**
CXCL1	Age at visit	0.0716	0.0475	0.5990
IL6	Age at visit	0.1015	0.0292	0.4604
IL6	Pre-mortem interval	−0.5339	0.0059	0.3745
IL8	Pre-mortem interval	−0.2469	0.0257	0.4604
ITGAM	Pre-mortem interval	0.1047	0.0253	0.4604

*Results shown are significant terms in multiple regression of plasma protein NPX on ABC-Braak score, age at visit, and pre-mortem interval. FDR calculations were based on n = 63 comparisons (21 proteins with 3 regression terms in model: NPX, age, and pre-mortem interval)*.

## Discussion

Tau pathology leads to senescence-associated transcriptomic changes (14, 15, 27) and subsequent vascular alterations in the mouse cortex, including transient capillary occlusion (27). Such intermittent episodes of capillary blockage arise from leukocyte adhesion in the endothelium, a phenomenon also reported in an APP-PS1 mutant mouse model of AD (75), as well as in chronic hypoperfusion (76) and traumatic brain injury (77, 78). This may be related to imbalanced capillary transit in AD, which can impair neuronal oxygenation even in the absence of gross reductions in cerebral blood flow (79, 80). Senescent endothelial cells secrete factors that attract peripheral leukocytes (81, 82) and upregulate surface leukocyte adhesion molecules (24, 73, 83, 84). Moreover, transcriptomic (85) and proteomic (86) analyses of human AD cortical microvessels have demonstrated upregulation of pathways related to senescence, including inflammation, leukocyte migration, and cell adhesion. However, this is the first study to directly examine the relationship between AD-related tau pathology and vascular senescence in both the cerebral vasculature and peripheral blood.

We report robust upregulation of the senescence- and leukocyte adhesion-associated genes CXCL8 (IL8), SERPINE1 (PAI-1), CXCL1, CXCL2, ICAM-2, and TIE1 in B3 cortical microvasculature. Interestingly, these genes are also involved in DNA damage response (DDR) signaling, a pathway that potently mediates cellular senescence (87–89). The pronounced upregulation of CXCL8 and SERPINE1 in B3 microvessels is notable, given their reported upregulation in senescent endothelial cells (23, 24, 90–92) and synergistic roles in leukocyte-endothelial adhesion (93). ICAM-2 also mediates leukocyte-endothelial adhesion in cerebral vasculature (94), and neurons upregulate ICAM-2 with the formation of tau NFTs (95). Tau burden and cerebrovascular pathology also appeared to additively interact with regard to microvascular expression of MAdCAM-1, a leukocyte adhesion molecule that is upregulated in the CNS following chronic vascular inflammation (96–98). Since MAdCAM-1 detection was variable across samples, future studies in a larger cohort are warranted to clarify the relationship between MAdCAM-1, tau pathology, and cerebrovascular pathologies.

One marker of cerebrovascular dysfunction is impaired blood-brain barrier (BBB) integrity, a phenomenon that has been linked to tau misfolding and aggregation (16, 99, 100). However, tau-independent BBB dysfunction is also observed with cognitive decline (101), possibly in relation to APOE ε4-associated BBB impairment in the hippocampus and medial temporal lobes (102). The BBB is comprised largely of endothelial cell tight junctions, supported by tight junction proteins such as OCLN, CLDN5, CDH5, and TJP1. We report here no change in gene expression of these four tight junction markers with tau progression in the AD cortical vasculature. This is a departure from previous reports of reduced cerebrovascular tight junction protein expression in AD (103) as well as the Parkinsonism–dementia complex of Guam (Guam PDC) tauopathy (104). However, Yamazaki et al. (17) showed that senescent endothelial cells exhibit aberrant tight junction protein localization without differences in overall tight junction protein expression, possibly reconciling this discrepancy. Furthermore, the assembly and localization of such tight junction proteins is largely influenced by post-transcriptional modifications (105).

Antemortem plasma protein expression did not associate with postmortem brain microvascular gene expression in this small sample, with the exception of NOS3. NOS3 is the endothelial source of nitric oxide (NO), a potent vasodilator that mediates vascular homeostasis and cerebral blood flow (106). While the association between gene and protein expression is generally tenuous, even within the same CNS cell type (107), future analysis in a larger cohort could determine the utility of NOS3 as a peripheral readout of cerebrovascular health. Of note, the temporal stability of a peripheral biomarker is an important consideration for its utility in studying the pathogenesis and prediction of AD. This is particularly relevant for PAI-1, which greatly fluctuated over time in both B1 and B3 plasma, possibly owing to its reported circadian variations (108) and cell surface binding after secretion (109). Furthermore, observed discrepancies in senescence-associated secretory phenotype (SASP) biomarker expression in cerebral vasculature vs. peripheral blood may be attributable to mismatches in transcriptional and translational upregulation.

One advantage of this study includes the preservation of the intercellular milieu in the neurovascular unit by isolating intact cortical microvessels. Additionally, the use of tau pathology scores, microvascular gene expression, and plasma protein levels from the same subjects enabled direct comparison across modalities. However, this study is limited by its small sample size, particularly in the plasma protein subgroup, with a bias toward AD samples. We included potential confounders such as sex, age, and cerebrovascular pathology in statistical models, though their roles in tau pathology and vascular dysfunction cannot be excluded. Additionally, AD commonly presents with other co-morbidities, including TDP-43 proteinopathy and Lewy body disease (110–112); future studies are warranted to compare tau-related vascular changes with and without such co-morbidities. Due to insufficient statistical power and a high degree of multicollinearity in this dataset, we did not adjust for APOE genotype nor for amyloid-beta burden, the effects of which also cannot be ruled out here. However, we note that the transcriptional changes shown in human AD cortical vasculature here are similar to those observed in the Tg4510 mouse model (15, 27), which exclusively over-expresses pathological tau. Future studies investigating individuals with high NFT burden but low amyloid accumulation, as seen in Primary Age Related Tauopathy (PART) (113), will be needed to confirm the specificity of these changes.

Looking forward, a larger sample size with evenly distributed pre-mortem intervals may reveal changes in the systemic vasculature that associate with cortical microvessel gene expression and tau pathology. Alternatively, rapidly advancing tau-PET neuroimaging represents a viable technique to track peripheral biomarker changes with tau accumulation in the AD brain. Tau tracer uptake correlates with the Braak stages of tau pathology progression in AD (114–116), and Ashton et al. (117) recently showed that tau-PET can be used to compare antemortem plasma protein expression and antemortem tau pathology. Real-time investigations of senescence-associated biomarker secretion in plasma and cerebral tau accumulation could better elucidate their relationship to vascular dysfunction in AD.

In conclusion, microvessels isolated from the human AD prefrontal cortex with extensive tau pathology upregulate genes involved in endothelial senescence and in recruiting leukocytes to the endothelium, which may contribute to AD-related vascular dysfunction and impaired cerebral blood flow. Future studies could identify peripheral biomarkers that are associated with vascular senescence and its relation to tau pathology in the human AD brain.

## Data Availability Statement

The original contributions presented in the study are included in the article/Supplementary Material, further inquiries can be directed to the corresponding authors.

## Ethics Statement

The studies involving human participants were reviewed and approved by Institutional Review Board of Partners Healthcare, Massachusetts General Hospital, Boston, MA. The patients/participants provided their written informed consent to participate in this study.

## Author Contributions

AB, SA, BH, and RB contributed to study concepts and experimental design. AB, MH, BC, and RB collected data. AB performed statistical analyses, with interpretation guided by input from BC, SD, and RB. MF provided access to biomaterials in the Massachusetts Alzheimer's Disease Research Center. AB drafted the manuscript, with critical revisions from BC, SD, BH, and RB for intellectual content. All co-authors reviewed the final manuscript and approved it for submission.

## Conflict of Interest

The authors declare that the research was conducted in the absence of any commercial or financial relationships that could be construed as a potential conflict of interest.
