# Supplementary material for: Cerebrovascular Senescence Is Associated With Tau Pathology in Alzheimer's Disease
Source: Front Neurol. 2020 Sep 16;11:575953. doi: 10.3389/fneur.2020.575953 (PMC7525127; doi:10.3389/fneur.2020.575953)
Supplement: Supplementary file 1 [file Data_Sheet_1.pdf]

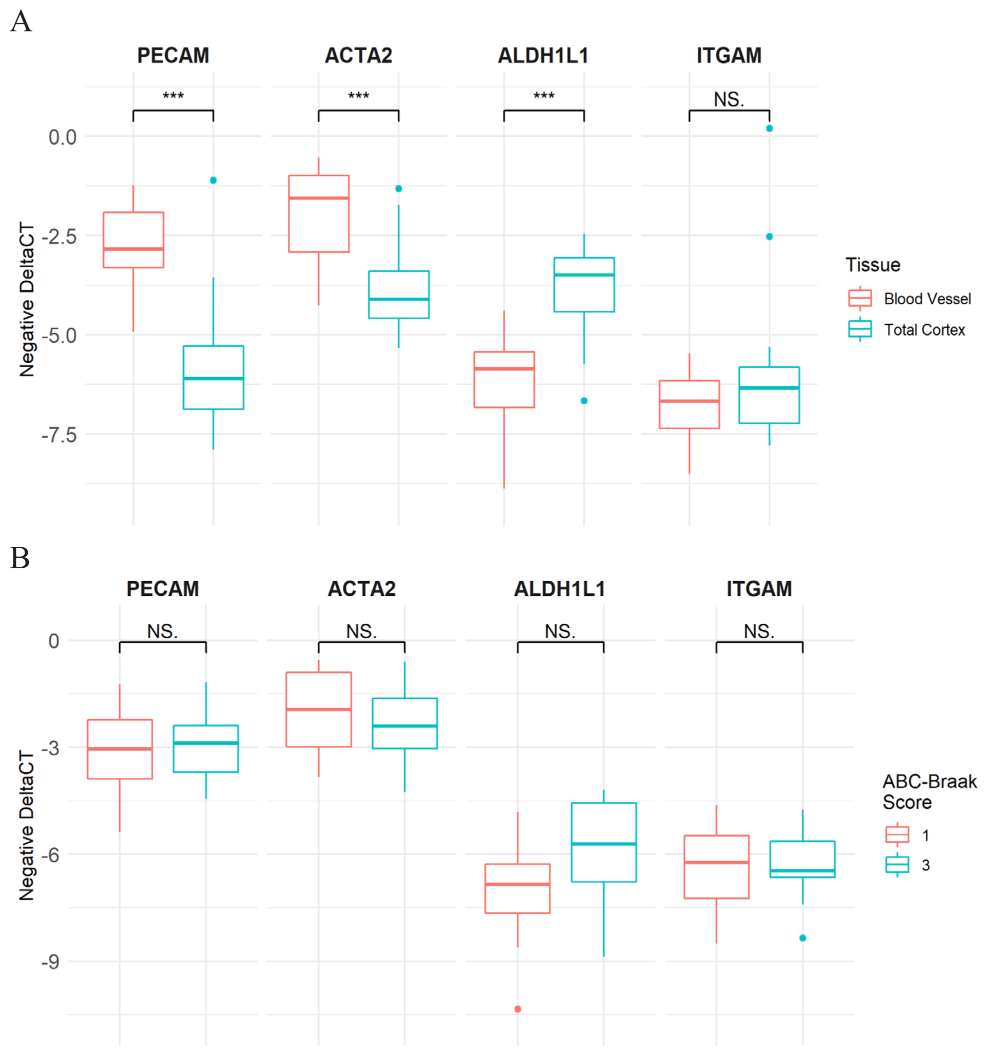

Supplementary Figure S1. Isolated PFC microvessels are enriched for endothelial and smooth muscle cells compared to total cortex. Note: for visualization, negative DeltaCT values are shown, representing the (negative) number of qPCR fluorescence cycles in between detection of the target gene and the reference genes.

(A) In n=8 B1 and n=8 B3 representative samples, the endothelial cell marker PECAM1 (CD31) and smooth muscle cell marker ACTA2 are significantly enriched in the isolated microvessels (***$p<0.001$). Astrocytes are under-represented in the microvessels compared with total cortex based on the astrocyte marker ALDH1L1 (***$p<0.001$), and no statistical difference was observed in microglial cell composition measured via the microglia marker ITGAM (CD11b).

(B) In all n=12 B1 vs. n=16 B3 microvessel samples, there is no statistical difference in endothelial cell, smooth muscle cell, astrocyte, or microglia composition. Across all samples, PECAM1 and ACTA2 expression is greater than that of ALDH1L1 and ITGAM, suggesting greater relative enrichment of endothelial and smooth muscle cells from the vasculature compared to astrocytes and microglia.

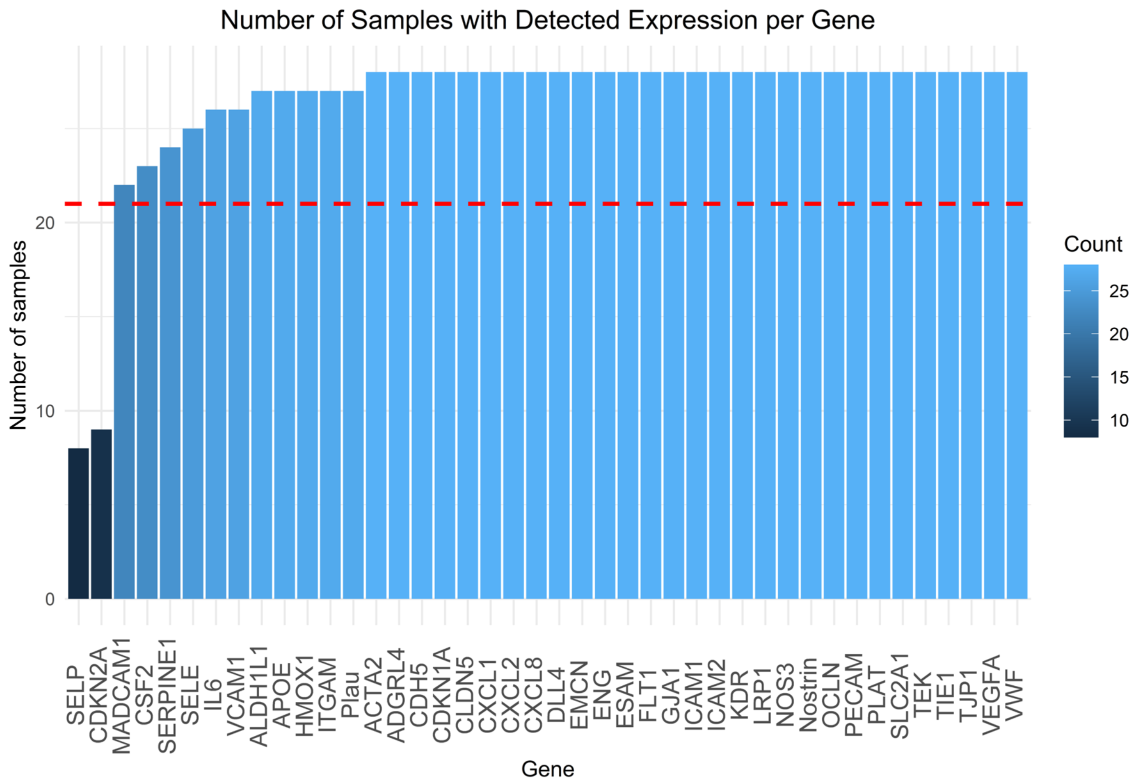

Supplementary Figure S2. Number of samples with detected expression per gene. The red dashed line indicates the 75% threshold of detected samples for a gene to be included in analysis, equating to a minimum of 21 out of 28 samples.

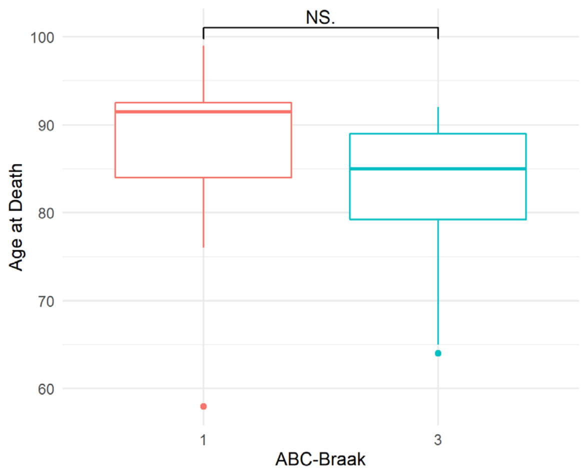

Supplementary Figure S3. Age at death is not different in B1 vs. B3 cases ($p=0.2544$, 95% CI: [-3.632, 12.965]).

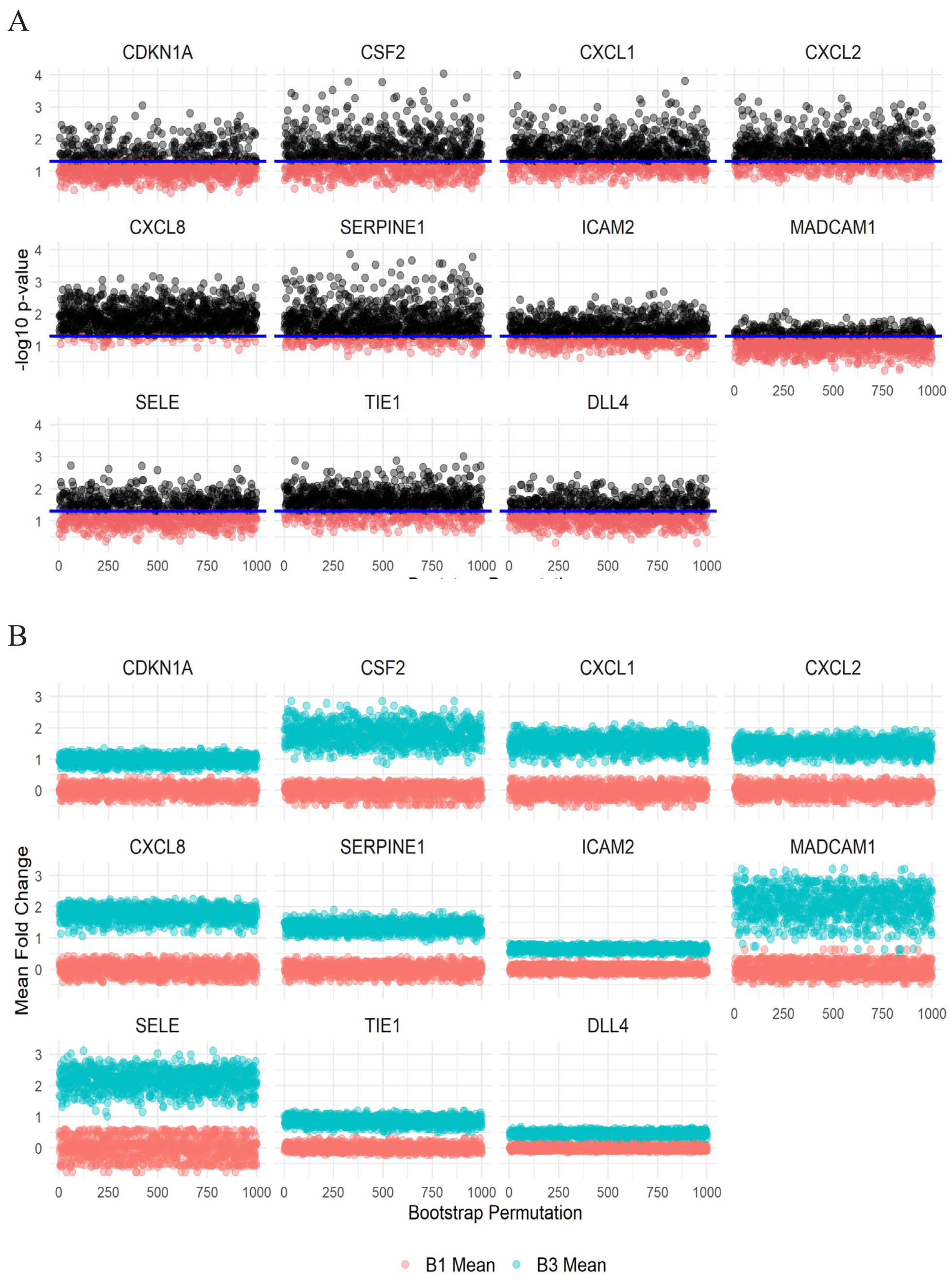

Supplementary Figure S4. Bootstrap permutation further supports upregulation of 11 genes, including 6 senescence-associated genes, in B3 PFC microvessels.

(A) Significance levels of t-tests from 1,000 bootstrapped sample permutations are plotted for the seven genes upregulated in B3 PFC microvessels. The blue line indicates the critical value for $\alpha=0.05$ in negative log base 10. Black points correspond to iterations with $p<0.05$ and red to iterations with $p>0.05$. CXCL8 yielded the most iterations with significant ($p<0.05$) results (93.9%), followed by SERPINE1 (79.7%), TIE1 (71.3%), CXCL2 (69.2%), ICAM2 (65.1%), CXCL1 (62.6%), CSF2 (54.7%), SELE (44.0%), DLL4 (43.3%), CDKN1A (31.0%), and MAdCAM1 (29.0%).

(B) The mean B1 and B3 log2 fold change values are plotted per gene for each of the 1,000 bootstrapped permutations of B1 and B3 samples.

A

ANOVA Term	p-value	BH-FDR
**ABC-Braak**	0.0170	0.2441
**Cerebrovascular Pathology**	0.0324	0.2979
**ABC-Braak:Cerebrovascular**	0.0076	0.2441

B

Tukey HSD Term 1	Tukey HSD Term 2	Log2 Fold Change Difference	Tukey HSD p-value	95% CI
**ABC-Braak/Cerebrovascular Pathology**	**ABC-Braak/Cerebrovascular Pathology**			
B3/Yes	B1/Yes	**5.120****	0.0020	(1.792, 8.449)
B3/Yes	B3/No	**4.189****	0.0073	(1.031, 7.346)
B3/Yes	B1/No	**4.435***	0.0142	(0.789, 8.081)
B3/No	B1/Yes	-0.932	0.7809	(-3.717, 1.853)
B3/No	B1/No	0.246	0.9961	(-2.991, 3.404)
B1/Yes	B1/No	-0.686	0.9362	(-4.014l 2.643)

Supplementary Figure S5. MAdCAM1 upregulation demonstrates interaction between ABC-Braak score and presence of cerebrovascular pathology.

(A) Preliminary ANOVA examining ABC-Braak score, cerebrovascular pathology, and the interaction of the two applied to all 40 genes demonstrates significant variance in each comparison before adjusting for multiple comparisons.

(B) Data presented are from Tukey's HSD post-hoc test from the ANOVA in S4A looking exclusively at MAdCAM-1, with p-values adjusted for multiple comparisons within the post-hoc calculations.

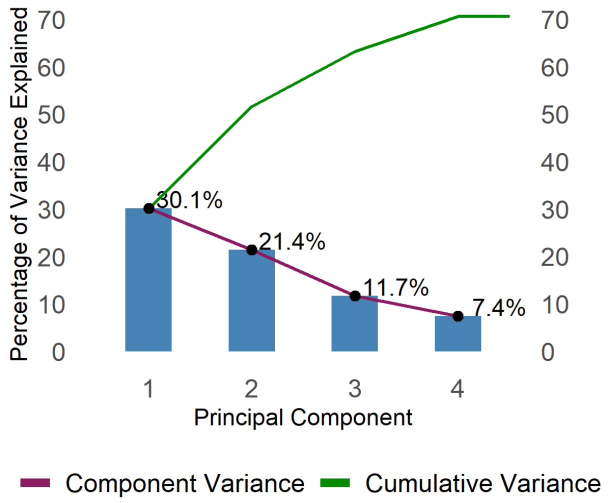

Supplementary Figure S6. Microvessel gene expression PCA captured more than 50% of sample variance in the first two components.

This Scree plot shows the proportion of sample variance explained in each orthogonal principal component. The purple line tracks the proportion of variance explained by each individual component, while the green line tracks the cumulative proportion of variance explained with the addition of each principal component. The first two components collectively explained more than half of the sample variance.

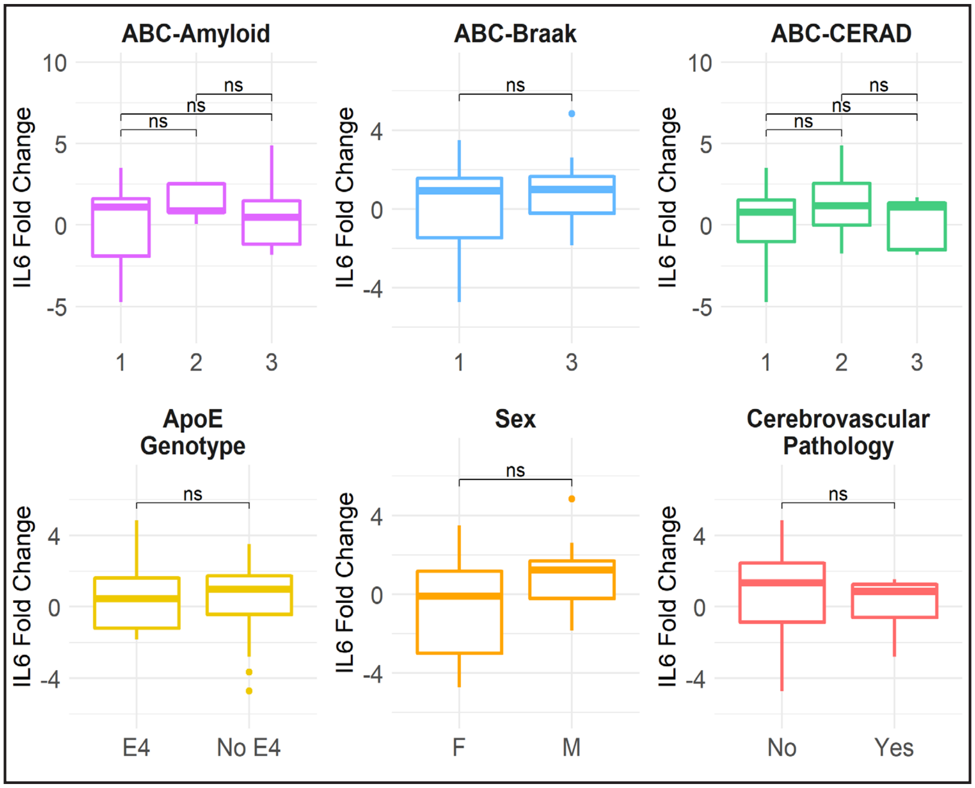

Supplementary Figure S7. IL6 expression (log2 fold change) is not different based on any neuropathological or clinical variable measured.

IL6 is not differentially expressed in PFC microvessels based on ABC-Amyloid, ABC-Braak, ABC-CERAD, ApoE genotype, sex, or presence of cerebrovascular pathology. n.s. = not significant in Tukey's HSD post-hoc analysis.

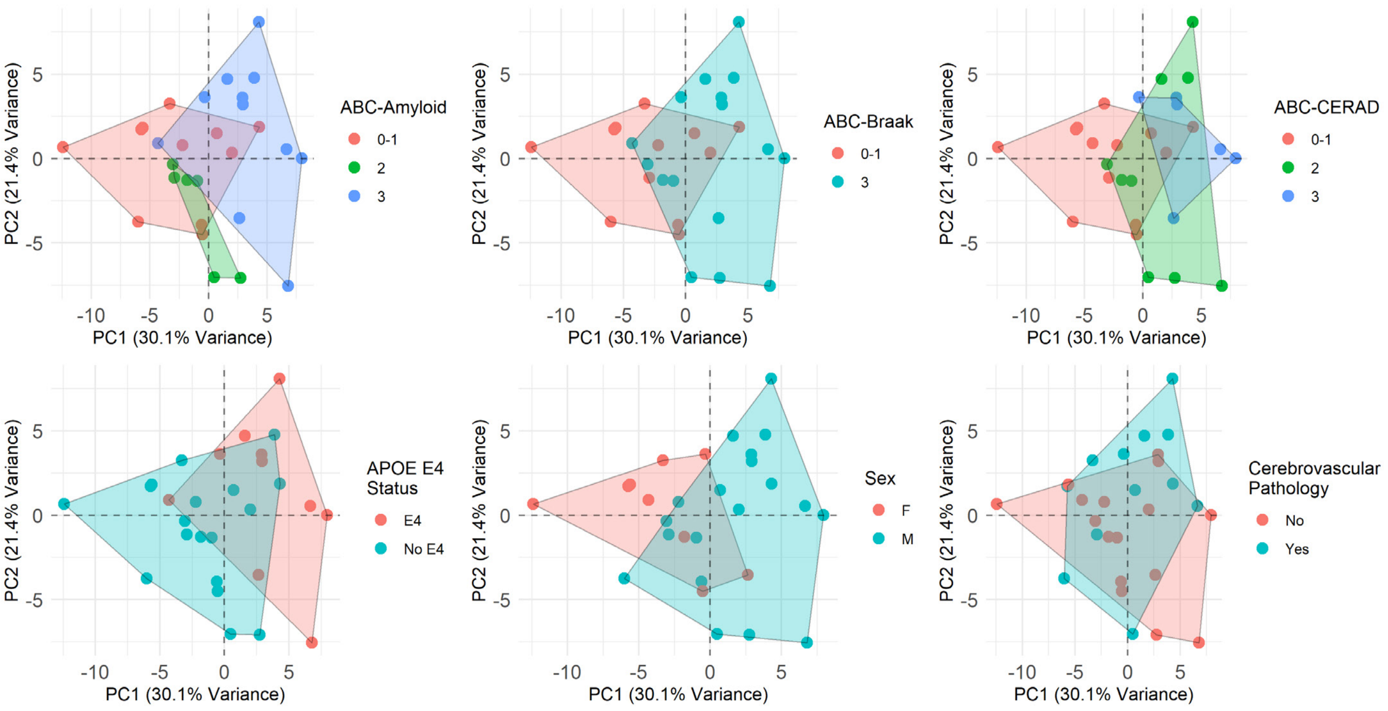

Supplementary Figure S8. Separation of subjects along PC1 vs. PC2 axes by neuropathological variables, APOE genotype, sex, and cerebrovascular pathology.

PC1 vs. PC2 scores from the gene expression PCA are plotted for each subject, with geometric encircling to show distribution of one neuropathological or clinical variable per plot.
